# Preclinical research models for endometrial cancer: development and selection of animal models

**DOI:** 10.3389/fonc.2025.1512616

**Published:** 2025-02-05

**Authors:** Yang Xue, Wei Shi, Bing Lun, Meilin Kan, Mengling Jia, Yuelin Wu, Li Yang

**Affiliations:** ^1^ Department of Obstetrics and Gynecology, The Third Affiliated Hospital of Zhengzhou University, Zhengzhou, China; ^2^ Zhengzhou Key Laboratory of Endometrial Disease Prevention and Treatment, Zhengzhou, China

**Keywords:** endometrial cancer, animal model, preclinical research, patient-derived xenograft model, humanized model, organoid

## Abstract

Endometrial cancer (EC) is the most common gynecological malignancy in developed countries, with rising incidence in recent years. Experimental animal models are crucial for studying the pathogenesis, advancing diagnostic methods, and developing new treatments. We review five main EC animal models. The use of spontaneous and chemically-induced models has decreased, with transgenic mouse and xenograft models becoming the most widely used. These models better simulate tumor molecular mechanisms and treatments, with the organoid-based patient-derived xenograft model (O-PDX) showing great promise in drug screening and personalized therapy. The application of humanized models remains limited due to technical challenges and high costs. In this review, we highlight the strengths and limitations of each model to guide researchers in their selection.

## 1 Introduction

Endometrial cancer (EC) is the most common gynecological malignant tumor in developed countries, and its incidence has been on the rise in recent years, bringing heavy medical burden to patients and hospitals (1, 2). The typical manifestation of EC is irregular vaginal bleeding, and postmenopausal patients often present with postmenopausal vaginal bleeding. These symptoms can attract patients’ attention early. Therefore, most patients with EC are diagnosed in the early stage, and the tumor is usually confined to the uterine body, leading to a relatively good prognosis through routine surgery. However, patients with advanced stage or metastasis usually lack effective treatment and have a poor prognosis (3). Currently, patients with early risk factors, such as advanced tumor, recurrence, or specific pathological types often opt for chemotherapy, which has been shown to be beneficial. Bevacizumab, as a monotherapy for molecular targeted therapy, has been included in the National Comprehensive Cancer Network (NCCN) recommended regimen since 2012 for patients with disease progression after prior chemotherapy (4). Researchers have tested the efficacy of bevacizumab in advanced endometrial cancer in real-world studies (5). Despite these advances, there is an urgent need for better and more effective treatments to improve outcomes for patients with advanced or metastatic disease. Some researchers suggest that defining early driver mutations can help distinguish different subtypes of endometrial cancer, facilitating the early detection of endometrial cancer or precursor lesions and guiding personalized treatment strategies. Some researchers suggest that defining early driver mutations can help distinguish different subtypes of endometrial cancer, facilitating the early detection of endometrial cancer or precursor lesions and guiding personalized treatment strategies (6). In 2013, The Cancer Genome Atlas (TCGA) project classified EC into four molecular subtypes through whole-genome analysis: (i) ultra-mutated endometrial cancer with mutations in the exonuclease domain of DNA polymerase epsilon (POLE), (ii) hypermutated endometrial cancer with microsatellite instability, (iii) copy-number-high endometrial cancer with frequent TP53 mutations, and (iv) the copy-number-low group of endometrial cancers (7). This classification has been widely adopted and proven to have a stronger prognostic impact than histopathological classification. It can predict patients’ response to chemotherapy, guide the selection of adjuvant therapies, and support the development of novel targeted therapies (8). Notably, it offers more opportunities and guidance for preserving fertility in young patients with endometrial cancer (9).Current research primarily focuses on clinical trials of targeted therapies and combination treatments as adjuvant therapies or for recurrent or metastatic endometrial cancer (10–13). However, these trials often require long durations and high costs (14). We need to utilize various preclinical models to provide direction for clinical trials.

The establishment of animal model plays an important role in the study of the occurrence and development of clinical diseases. The successful establishment of an ideal animal model helps us to understand the pathogenesis and biological behavior of EC, and becomes an important means to develop relevant targeted drugs and achieve early treatment, providing more treatment opportunities for patients with advanced endometrial cancer. However, there is no comprehensive review that summarizes the various preclinical animal models of EC, which include rabbits, cows, pigs, and the most commonly used mice. Each animal model has its own value in studying disease progression and treatment, but these models have not been systematically reviewed in the literature. By searching databases such as PubMed, Web of Science, Embase, and CNKI with the keywords “endometrial cancer” and “animal model”, we have summarized five commonly used EC animal models: spontaneous, chemically-induced, transgenic mouse, xenograft, and humanized model. In subsequent research, we use these five models as search terms to obtain more comprehensive information. These include the earliest spontaneous and chemically induced models, genetically modified mouse models that have become common with advancements in genetic engineering technology, xenograft models (including the new patient-derived xenograft models and patient-derived organoid models) that better study the tumor microenvironment, and the latest humanized mouse models. Each model has its own strengths and weaknesses, and while they can complement each other, they are not entirely interchangeable. We have summarized the advantages and limitations of these five models (**Table 1**) with the aim of providing guidance for preclinical researchers.

**Table 1:** Summary of the pros and the cons of animal models of endometrial cancer.

Animal Models of EC	Pros	Cons	Research directions
Spontaneous	No special treatment required; Economical and convenient, no need to ensure a sterile environment; Immune system integrity; Representation of the whole process of cancer development;	Long culture cycle; Low incidence and unstable;	Exploration of risk factors; Observation of the development process; Therapeutic research; Toxicological study; Hormone carcinogenesis;
Chemically induced	Similar histopathology; High incidence; Simple operation;	Unclear genetic background; Long time to induce;	Carcinogenesis caused by chemical exposure; Carcinogenic molecular pathway mechanism of estrogen; Combine with other models to overcome some limitations;
Genetically engineered	Immune system existence; Specific gene mutation;	Difficult operation; expensive; Low tumor mutation load;	Molecular pathway mechanism; New molecular therapeutic targets;
Xenograft	Low cost; Short culture cycle; Predictability of tumor growth; Easy to observe;	Immune deficiency; Limited similarity to human;	Evaluation on drug efficacy and sensitivity; Biological characteristics of cancer; Biomarkers and mechanisms of drug resistance; Immunotherapy research;
Humanized	Related to the human immune system; Advantages in immunotherapy research;	Technically difficult; High cost; Incomplete reconstruction of the human immune system;	Tumor-immune system interaction; Immunotherapy;

## 2 Animal models of EC

### 2.1 Spontaneous models

The natural incidence of endometrial cancer is notably high in certain animals, and these species can be used as models to observe the natural occurrence and development of EC *in vivo* and explore the factors affecting the development of tumors. G Vollmer (15) introduces four rat strains with abnormally high incidence of endometrial adenocarcinoma, namely Han: Wistar rat, Donryu rat, DA/Han rat, and BDII/Han rat. Among these, the first two rat strains show similar but relatively low rates of EC during their natural lifespan, 39% and 35.1%, respectively. The tumor model in the Donryu rat is considered to be hormone-dependent, and some of its pathogenesis characteristics have been discussed as potential risk factors for promoting the development of human EC. In addition, this rat strain is frequently used to observe the development of endometrial cancer (16) and to conduct therapeutic studies (17). Most research on the Donryu rat model has focused on the decade after its discovery. However, in recent years, research on this strain has decreased, possibly because of its low tumor incidence and long cultivation cycle. It typically develops endometrial hyperplasia at 8 months of age, with an increase in incidence and severity, eventually progressing to adenocarcinoma at around 15 months (16). With the emergence of better hormone-dependent models that shortened the culture cycle, the application of the Donryu rat model has gradually declined. On the other hand, the Han: Wistar rat model is primarily used in toxicological studies to explore the effects of drugs on the incidence of EC (18–20). The incidence of EC in the DA/Han and BDII/Han rat strains is higher, with rates of 60% and 90%, respectively. The near-bred DA/Han and BDII/Han rats are considered an appropriate model for studying hormonal carcinogenesis (21), as they primarily succumb to hormone-dependent endometrial adenocarcinoma. The tumor phenotype in this model closely resembles that of human tumors, with a high probability of metastasis, making it suitable for intervention studies. In addition, a continuous transplantable tumor line, EnDA (22), can be established from these tumors, and the RUCA-I cell line (23) can be cultured to form tumors that stably express estrogen receptors (ER) and progesterone receptors (PR), exhibiting high tumorigenic potential. Both EnDA and RUCA-I cell lines can be transplanted into syngenic DA/Han rats, negating the need for immunodeficient mice which have to be kept in germ-free environments. Xenograft models established with this cell lines are often used to study the excitatory and antagonistic effects of estrogen and anti-estrogens on the development of hormone-dependent endometrial carcinoma (23, 24).

In addition, rabbits and cattle also exhibit higher spontaneous rates of EC. Most spontaneous endometrial carcinomas in rabbits are multiple and bicornual. Histologically, the majority are adenocarcinomas, with significant variability in the degree of differentiation (25). In cattle, spontaneous endometrial carcinoma is typically solitary, and histologically, it is often adenocarcinoma, frequently accompanied by the formation of dense fibrous tissue. However, due to the higher costs of these animals, longer culture cycles, and less convenient tumor observation, their use is less common compared to rat models.

### 2.2 Chemically induced models

Exposure to various chemicals can lead to abnormal cell proliferation and differentiation, resulting in the occurrence of advanced cancers, particularly in hormone-responsive tissues (such as breast, ovarian, endometrial, and prostate cancers). The incidence of cancer in early development after exposure to certain chemicals will be significantly increased. The first chemical to be studied in relation to EC was diethylstilbestrol (DES). DES was initially used to prevent miscarriage, but neonates exposed to DES in the perinatal period showed numerous abnormalities in reproductive tract development and function (26). This led to the establishment of several laboratory rodent models (27–29), which demonstrated that the incidence of EC in adolescence following early-life exposure to DES exceeded 90%. Moreover, the incidence increased with the increasing dose of DES, even at very low doses (30). Since then, chemically induced animal models have been widely used in various experimental studies. Subsequent studies have demonstrated that exposure to chemicals such as endogenous/exogenous estrogens, endocrine-disrupting chemicals (EDCs) (31), N-ethyl-N’-nitro-N-nitroguanidine (ENNG) (32), benzotriazole (BTR) and its derivatives (33), and tetrabromobisphenol A (TBBPA) (18) can promote the development of EC. Among these, the histopathology of tumors induced by exposure to both endogenous and exogenous estrogens closely resembles that of human tumors, and significant progress has been made in therapeutic research related to these models (34–37). Previously, it was believed that exposure to chemical carcinogens led to unpredictable genetic changes. However, with the advancement of molecular testing technologies in recent years, studies have increasingly focused on how chemical exposure alters molecular pathways involved in EC development. These chemicals mostly interact with or mimic estrogen pathways *in vivo* (38). Key factors associated with these changes have been identified, including SIX1 (39), CTBP1 (33), cytochrome P450 1B1 (40), PTEN (41), Erα, PCNA, p53 (42), COX-2 (43), and Wnt-7a (44). These studies have provided valuable insights into the transcriptional alterations that accompany the development of estrogen-driven EC, revealing potential carcinogenic mechanisms.

Due to the limitations of chemically-induced models—such as significant tumor differences from human cases, long induction times, and low tumor formation rates- other models are now preferred. However, the combined application of chemical exposure models and other models still holds certain value. For instance, combining them with spontaneous models or genetically engineered mouse models can shorten the culture cycle, or they can be used to assess the carcinogenic potential of various genotoxic chemicals based on the genetically engineered mouse models (45). To some extent, this approach can overcome the limitations of individual models and enhance experimental efficiency.

### 2.3 Genetically modified models

In the past few decades, the development of transgenic and gene targeting technologies has facilitated the development of genetically modified models (GMMs), and tumor biology studies using transgenic animals. Common genetic modification methods include homologous recombination, CRISPR-Cas9 technology, viral-mediated gene transfer, and transgenic techniques. Homologous recombination is the most classic method for constructing genetically modified mice (46). By designing a vector carrying the target gene mutation and introducing it into embryonic stem cells, the foreign gene or mutation is incorporated into the mouse genome through homologous recombination, generating transgenic or knockout mice. CRISPR-Cas9 technology is a widely used gene editing tool in recent years. This technology uses specific RNA to guide the Cas9 protein to cut DNA at target sites, enabling gene mutations or insertion of foreign genes with high efficiency and precision (47). Viral-mediated gene transfer typically uses viral vectors such as adenoviruses or lentiviruses to deliver foreign genes into the mouse body (48), which is particularly advantageous for constructing high-expression models. Transgenic techniques involve directly injecting foreign genes into fertilized eggs or early embryos (49). The injected genes integrate into the genome during embryo development, and this method is commonly used to create models with overexpression of certain genes either throughout the body or in specific tissues. Due to the physiological similarities between mice and humans, as well as the resemblance in mutation phenotype to human genetic diseases, mice have become an ideal model for studying human tumor biology. In addition, the stable genetic background, rapid reproduction, and large litter size of mice make them ideal candidates for transgenic research. This section will focus on introducing various genetically engineered mouse models of EC. The advantage of GMMs over other models lies in their ability to elucidate initial mutations, providing valuable insights into the specific proteins and signaling pathways involved in the development of EC. These models accelerates our understanding of how individual genetic lesions contribute to tumorigenesis, facilitate preclinical trials of new therapeutic targets, and are particularly crucial for advancing molecular targeted therapies.

#### 2.3.1 Models of common genetic abnormalities

Mice with endometrial-specific PTEN or p53 deletion are considered ideal models for studying EC. In human type I EC, the most common genetic mutation is the loss of function of PTEN, a tumor suppressor (50), which is observed in 30%-80% of type I EC cases and 20% of complex atypical hyperplasia. Studies have shown that PTEN mutations precede the appearance of histologically identifiable proliferative lesions, making PTEN loss an early event in the multi-step process leading to EC (51). P53 inactivation is a major driver of most serous cancers, some high-grade endometrioid cancers, and many uterine carcinosarcomas (52). It is associated with aggressive behavior and an increased risk of recurrence, and is considered a late event in disease progression (53). The conditional p53 knockout model accurately reflects the progression of human tumors from precursor lesions to invasive type II EC. This model is similar to human type II endometrial tumors, exhibiting strongly expressed nuclear KPNA2 in some adenocarcinoma models. It is speculated that the androgen receptor may serve as the target nuclear receptor of KPNA2 shuttle (54), making this model suitable for studying the shuttle mechanism of KPNA2. In addition, deficiencies in tumor suppressor genes such as BRAC2 and ARID1A, as well as amplification of oncogenes such as ERBB2, KRAS, CCNE1 and MYC, are also common genetic abnormalities found in EC. These factors can be used to establish EC tumors in GMMs. Building on these common genetic factors, researchers continue to develop new epithelial-specific expression promoters to induce gene conditional knockout (51, 55), further investigating the role of various signaling pathways and molecular signals *in vivo*. This ongoing research has greatly enriched our understanding of the molecular mechanism underlying the development of EC.

#### 2.3.2 Other GMMs

The Mig-6 conditional knockout model (56) has demonstrated that Mig-6 plays a crucial role as a mediator in steroid hormone signal transduction in the uterus, inhibiting tumor growth by enhancing the antagonism of P4 to E2. This model provides a reliable model for studying the pathology and hormone sensitivity of EC (55). The LKB1 conditional knockout model{sp} (51) {/sp}induces the formation of invasive endometrium carcinoma with 100% penetrance, often causing symptoms due to local invasion and spread, but rarely distant metastasis. These features closely resemble human EC. This model can be used to explore the combined lethal effect of mTOR inhibition and LKB1 deficiency, as well as to investigate the impact of long-term rapamycin administration on tumor growth and the mechanisms of resistance in cases of relapse. Sullivan et al. (57) evaluated the efficacy of metformin using a combined defect model of LKB1 and p53, which best reflected high-grade EC and provides a preclinical model for therapeutic clinical trials of metformin in EC patients. The SOX9 overexpression model (58) revealed the formation of cystic gland structures in the endometrium, with histological analysis showing that these structures were morphologically similar to endometrial hyperplasia and polyps. This model deepens our understanding of the molecular events at each stage of EC progression. The FOXA2 conditional expression model (59) can be used to analyze the signaling hierarchy between FOXA2 and other genes, facilitating the screening of drugs that directly target FOXA2, which could accelerate the development of new therapies. Tirodkar et al. generated triple transgenic MUC1^+/-^loxP-STOP-Kras^G12D/+^Pten^loxP/loxP^ (abbreviated as MUC1KrasPten) mice (60), which express physiologic levels of human MUC1 as a self-antigen under steady-state conditions. KrasPten-driven uterine tumors express human MUC1 and trigger spontaneous anti-MUC1 antibodies. The researchers observed that the entire reproductive tract epithelium exhibited similar genetic changes at the same rate, but the tumor microenvironment appeared to be a key determinant of tumor grade and survival. The MUC1KrasPten mouse model was the first preclinical model with normal immune function and *in situ* expression of human MUC1. However, its limitation is that the model does not show 100% penetrance, and the tumor-bearing mice do not exhibit signs of endometrial hyperplasia. The luteinizing hormone receptor (LH-R) overexpression model (61) can be used to explore the carcinogenic role of the LH/LH-R axis in the development and progression of EC. Targeting the LH/LH-R axis may provide a valuable therapeutic strategy. Maru et al. (62) reviewed various *in vivo* and *in vitro* transgenic models, describing recent advancements in the exploration of the molecular mechanism of EC. They also analyzed several different transcriptional promoters used for gene knockout, providing useful references for the development of new GMMs in EC.

These different GMMs have been instrumental in studying various molecular pathways, further enhancing our understanding of the molecular mechanisms underlying the initiation and progression of EC. Notably, the knockout and overexpression of *p53* represent two major subtypes in the new molecular classification of EC, which are closely linked to patient prognosis. This model holds significant promise for advancing future research on targeted therapies.

### 2.4 Xenograft models

The classic EC xenograft models are established by injecting human tumor cell lines or implanting solid tumor blocks under the skin, *in utero*, or in other sites in immunodeficient animals, which helps overcome interspecies immune rejection.

The typical heterotopic xenograft model involves subcutaneous injection of tumor cells. This method is relatively simple, has a short incubation period, and the transplant success rate can be as high as 100%, making it the most commonly used modeling approach. The most frequently used cell line is Ishikawa, which is estrogen-sensitive and of great significance for studying endocrine therapies. It can also be utilized to test various combination therapies. There are also many cell lines with low estrogen sensitivity, such as HEC-1A, HEC-1B, RL95-2, KLE, JEC and AN3CA. In addition to injecting cell lines, tumor tissue blocks can also be implanted. The tissue blocks are typically cut to 1-2 mm in size to ensure adequate blood supply to the tumor center and to promote normal growth. The subcapsular renal implantation model (63) and peritoneal implantation model address the limitations of the subcutaneous transplantation model, such as poor blood supply, lymphatic drainage, and inability to form invasion and metastasis. These models provide a microenvironment conductive to tumor growth and can simulate drug administration routes for evaluating the efficacy of anticancer drugs during cancer development. Zheng Jing et al. (64) improved the classic subcutaneous transplantation model by proposing a mixed inoculation of Matrigel and high-concentration Ishikawa tumor cells. This method significantly shortened tumor formation time and increased the tumor formation rate. The interaction between tumor cells and the transplantation microenvironment is of great significance to the transplantation process. Therefore, the microenvironment in orthotopic xenograft is more conducive to simulating the growth environment of human EC and establishing tumor biological characteristics that more closely resemble human EC. While many successful *in-situ* transplantation experiments (26, 65–67) have been conducted, their main limitation lies in the difficulty of transplantation procedure. In the rabbit model of prostate cancer, some researchers have proposed to use transabdominal ultrasound-guided injection of cancer cells (68). This method shortens the recovery time of rabbits compared to conventional laparotomy. This approach can be adapted for animal models of EC to improve transplantation techniques and enhance efficiency.

In recent years, patient-derived xenograft (PDX) models have emerged as a powerful tool for cancer research. These models involve the surgical excision of tissue from patients, which is then implanted into immunodeficient mice. Successful PDX models have been established for various cancers, including lung, gastric, colorectal, breast, prostate, and EC. The EC PDX model, in particular, closely mimics the histopathological features of human EC, maintaining the tumor’s molecular characteristics, primary tumor heterogeneity, and tumor microenvironment. This makes PDX models valuable for evaluating the efficacy of novel therapies and identifying therapeutic response biomarkers (69–71), offering high predictive value for treatment outcomes. However, the challenges associated with PDX models include the relatively long time required to establish them, the complexity of the procedure, and the low success rate of primary transplantation. Improvements in these areas are still needed to enhance the efficiency of PDX model generation. Organoid models are three-dimensional *in vitro* cultures that replicate the structure and function of the organ from which they are derived. These models can be designed by introducing cancer-associated genes into normal organoids using tumor tissue specimens or CRISPR-based genetic modifications (72). Patient-derived organoid (PDO) models offer several advantages, such as the ability to simulate the tumor microenvironment and cell-extracellular matrix (ECM) interactions, as well as preserve tumor heterogeneity and diverse cell populations. PDO models have become widely used in basic cancer research, drug testing, and personalized medicine (73–75). By transplanting PDOs as xenografts into immunodeficient mice, organoid-based patient-derived xenograft (O-PDX) models can be established, which replicate the mutation characteristics of the primary tumor, simulate disease progression, and even exhibit metastatic lesions. Researchers like Yoshiaki Maru (76) and Berg HF (77) have successfully developed orthotopic O-PDX models for EC, while Jingyao Chen et al. (78) created a novel mouse EC model using genome editing and organoid formation, termed the organoid-initiated precision cancer model (OPCM). This model allows for the targeted mutation of genes like p53 and PTEN, as well as MYC overexpression, enabling researchers to study the effects of specific genetic alterations on tumorigenesis and use it for preclinical drug screening. Despite their advantages, the current challenges for organoid models include improving their growth efficiency, reducing generation costs, standardizing culture protocols, and expanding co-culture models to better replicate the internal TME. The PDO model, in combination with the O-PDX models, offers a platform that closely mirrors human physiology and is widely used in experimental and preclinical drug research. However, the suppression of immune function in both heterotopic and orthotopic xenograft models remains a significant limitation, making them less suitable for studies on tumor immunology.

### 2.5 Humanized models

Xenograft models have proven to be highly valuable in laboratory research, but their main limitation lies in their inability to accurately replicate a functional human immune system. To overcome this constraint, animal models with human immune systems have been developed in recent years, enabling the testing of immunotherapies and providing excellent preclinical platforms for drug development and novel immunotherapeutic strategies. These models, known as “humanized mice”, are immunodeficient mice transplanted with functional human cells or tissues. The focus of humanized models is on reconstructing the human immune system, which is achieved by injecting human fetal thymus, umbilical cord blood cells, adult peripheral blood stem cells, or adult peripheral blood lymphocytes into immunodeficient animals, typically via veins, the abdominal cavity, or the spleen (79, 80). Severe combined immunodeficiency (SCID) mice are most commonly used for this purpose. Humanized mice are advantageous over conventional immunodeficient mice, as they allow for more efficient establishment of the human immune system, improved implantation success, and enhanced susceptibility to infection. The intraperitoneal injection of human peripheral blood lymphocytes is one of the simplest and most cost-effective methods for generating these models. Several humanized mouse models of various cancers, including lung, breast, liver, and colorectal cancers, have been successfully established. These models are capable of replicating normal human immune system responses, demonstrating robust remodeling of human immune cell, and producing antigen-specific antibodies. This greatly facilitates research into tumor-immune system interactions (80) and is crucial for advancing the understanding of the immune response and tumor microenvironment *in vivo*. Another promising avenue is the development of PDX models in humanized mouse models. By transplanting both the human immune system and tumor tissues into these mice, researchers have created animal models that combine immune reconstitution with the implantation of patient-derived tumors. These models offer a more accurate simulation of the human tumor microenvironment and hold great potential for establishing more predictive preclinical models, particularly for studying human EC (81). For instance, Yuyo Ka et al. (82) transplanted human intestinal microflora into sterile humanized mice, creating a dual-humanized mouse model. In this model, the number of human CD3^+^ T cells increased slightly, representing a promising new approach to studying cancer immunology. However, the study primarily focused on the interaction between human immunity and the gut microbiome, and this dual-humanized model has not yet been applied to the development of cancer models. We can anticipate the establishment of a dual-humanized mouse model of EC for the first time, which would offer a unique opportunity to study the interactions between tumor immunity and the immune system.

While humanized models hold great promise for tumor immunotherapy research, a successful humanized EC model has yet to be developed. However, the experience gained from other cancer models can serve as valuable lessons and guide the development of a humanized EC model. This represents a highly promising research direction that could significantly contribute to our understanding of EC immunotherapy. Ultimately, it holds the potential to advance the field toward truly “personalized” medicine, offering more tailored and effective therapeutic strategies for EC patients.

## 3 Conclusions

The establishment of the animal models for EC has provided immense value in advancing our understanding of its pathogenesis and in developing new therapeutic strategies. To date, several commonly used animal models have been proposed. The spontaneous and chemical induction models, which were primarily focused on over 20 years ago, remain integral to cancer research due to their unique applications and the insights they provide into tumor initiation and progression. The development of genetic engineering models has paralleled advances in transgenic technology, and in today’s rapidly evolving scientific landscape, these models continue to hold promise for exploring the molecular mechanisms underlying cancer development. Among the most widely used models is the xenograft model, which has proven to be a powerful tool in cancer research. PDX and O-PDX models, both derived from xenograft technology, have become essential in evaluating drug sensitivity and effectiveness, with significant prospects for future research and clinical application. However, challenges remain in improving the success rates and efficiency of model establishment, and the exploration of better modeling techniques is still needed. Humanized models have opened up new avenues for studying the interaction between the human immune system and tumors. By combining the PDX model with human immune system reconstruction, humanized models allow for the joint study of immune response and the tumor microenvironment. While the establishment of humanized models has been successfully achieved in other cancers, applying this model to EC still requires further research and refinement. The combination of molecular profiling, genetic engineering, and immune profiling in gene-engineered and xenograft mouse models is crucial for advancing our understanding of the tumor microenvironment and holds great promise for the development of novel immunotherapies. These therapies could potentially improve the prognosis of patients with recurrent or metastatic EC, a group that currently has limited treatment options. Researchers must understand the advantages and limitations of each animal model, selecting the most appropriate model for specific research purpose. This selection is crucial to the value and success of experiments. Furthermore, there is an ongoing need to refine existing models and develop new, more sophisticated humanized models to fill existing knowledge gaps, particularly in the context of advanced, metastatic, or rare pathological subtypes of EC. Such advancements will help provide more personalized treatment options for patients, particularly those with tumors that exhibit resistance to conventional therapies.
